# Everybody's Page

**Published:** 1892-07-16

**Authors:** 


### Everybody's Page.

The P. and O. and Orient Steamship Companies are granting special concessions to intending visitors to the Inter-Colonial Medical Congress, which is to be held at the end of September, in the Medical School of Sydney University, and as the railways are quoting special low rates also, there will no doubt be a large gathering.

How absolutely necessary complete isolation is in cases of scarlet fever and small-pox is shown by what happened recently in Paris. A child was sent back to school before having recovered completely from scarlet fever, with the painful result that 150 cases shortly broke out in the school, followed by 18 deaths. Here is clearly a case of preventible disease which should never have been permitted to occur.

The postal facilities and service at the World's Fair at Chicago are to constitute a part of the United States Government exhibit, and no doubt every effort will be made to show the world what can be done in this direction. Here is a golden opportunity for St. Martin's-le-Grand to take some hints, for there is plenty of room for improvement in our postal service, which in many particulars is painfully wanting, and lamentably behind the times.

It is high time Boston looked to her literary laurels. It must be somewhat galling to her pride that she has a rival, and no unworthy one, in the home of pork. Chicago, according to a contributor to the *Atlantic Monthly*, has already accumulated a public library of 175,000 books, which is being increased at the rate of 20,000 volumes annually. A purchase of one hundred thousand volumes *en bloc* seems to be a trifle to this go-ahead city which means to show the world in 1893 what enterprise can do.

Cholera may or may not reach our shores, but though the surroundings generally in this country are not particularly alluring to it, isolated cases will probably occur in some of our seaports and larger towns. Whatever happens, there should be no scare. for prevention is especially efficacious with cholera. Those who take ordinary precautions, live temperately amid cleanly surroundings, avoid excessive exercise of mind or body, boil their milk and water, boil the water in which they wash their vegetables and salads, and refrain from stone-fruit, should have no cause for fear.

Many years ago, so says Mr. Walter G. Walford, a consignment of skulls was received at the Anatomical Museum at Cambridge. These skulls had been excavated from barrows principally in Dorsetshire, and were considered by scientists to be those of Ancient Britons. The remarkable feature of these skulls, which were almost all those of adults, was the perfect preservation of the teeth; and Mr. Walford concludes, no doubt correctly, that "in the primeval days, when these skulls belonged to living people, dentists could have found next to nothing to do." Oh, happy days, and lucky Ancient Britons, but then they were as simple in their living as they were in their attire, while we do everything conceivable to make the dentists' lot a happy one, and to encourage the propagation of his species!

If Miss Ormerod and the British farmers who owe so much to her have their way the sparrow is doomed. But this aggressively impertinent bird has so often been threatened that we still believe his end to be far off. Miss Ormerod has declared in no faltering accents that the sparrow is a curse to British agriculture, yet such is the audacity of this chirper that we doubt whether the knowledge of the evidence being collected against it would help to subdue its spirit and mend its wicked ways. In the matter of energy the British farmer might take a lesson from his great enemy.

Greece has always been associated in our minds with romance. Can it then be true that there is one spot in that poetic country where love and romance are unknown quantities? In Kaso, one of the islands of Greece, a contributor to the *Eastern and Western Review* tells us courtship, as we understand it, is unknown. Everything is arranged by the parents, who hurry off their victims to the altar whether "Barkis is willing" or no. Possibly the result of this matter of fact arrangement may be all that can be wished, and the courtship begins after marriage; whereas in most countries, we are given to understand, it generally ceases immediately after the consummation of that event.

Reports of school inspectors are often amusing and never fail to be instructive, and that of Mr. Airy, on the Birmingham district, is no exception. It is refreshing to find that the adoption of common sense in dealing with the education of infants has had its merited reward. The result is unmistakeable happiness amongst the children whose afternoon's work is made to tax the brain as little as possible, and Mr. Airy's experience teaches him that, "wherever the abandonment of headwork in the afternoon is greatest," there "the advance in knowledge is most marked." An excellent feature in the system adopted in this district, which might advantageously be copied everywhere, is the spending of the latter part of the afternoon amongst the younger children in relating stories. Time so spent is never wasted.

The National Lifeboat Institution have drawn timely attention to their series of directions for restoring those who apparently are drowned. Now that the boating and bathing season is at its height, opportunities, unfortunately, are sure to occur for putting these directions to practical use. An interesting instance of successful restoration, where doctors had pronounced life to be extinct, is reported from Normandy by the *Daily News* correspondent. Professor Laborde, whilst fishing, heard of an accident to a young man who was reported by two doctors, to be dead, and at once proceeded to the scene of the fatality. He found that the heart had stopped, at least to all appearances, but he seized the root of the man's tongue and drew it forward violently by successive jerks so as to excite the reflex action of the breathing apparatus. At the end of a few minutes a slight hiccough showed life was not extinct, and the professor, who continued with the usual restorative means, supplemented by the rubbing of the chest with hot towels, saved a life which, but for his fortunate presence, would have] been irretrievably lost.

